# Research of the Active Components and Potential Mechanisms of Qingfei Gujin Decoction in the Treatment of Osteosarcoma Based on Network Pharmacology and Molecular Docking Technology

**DOI:** 10.1155/2022/7994425

**Published:** 2022-11-23

**Authors:** Qingying Yan, Jiewen Yang, Yongwei Yao, Zhen Jia, Yiqing Wang, Miao Cheng, Xiaobo Yan, Yefeng Xu

**Affiliations:** ^1^Department of Oncology, Hangzhou Third People's Hospital, Hangzhou, China; ^2^Department of Oncology, Affiliated Hangzhou Dermatology Hospital, Zhejiang University School of Medicine, Hangzhou, China; ^3^Department of Orthopedics, The Second Affiliated Hospital, School of Medicine, Zhejiang University, Hangzhou, China

## Abstract

**Aim:**

Qingfei Gujin Decoction (QGD) has been shown to be effective against osteosarcoma. This research was aimed at investigating the main active ingredients and potential mechanisms of QGD acting on osteosarcoma through network pharmacology and molecular docking techniques.

**Methods:**

The active ingredients and targets of QGD were screened from the TCMSP database, and the predicted targets were obtained from the PharmMapper database. Meanwhile, the targets of osteosarcoma were collected using OMIM, PharmGKB, and DisGeNET databases. Then, GO and KEGG enrichment analyses were performed by RStudio. PPI and drug-ingredient-target networks were constructed using Cytoscape 3.2.1 to screen the major active ingredients, key networks, and targets. Finally, molecular docking of key genes and their regulatory active ingredients was performed using AutoDockTools 1.5.6 software.

**Results:**

38 active ingredients were collected, generating 89 cross-targets; quercetin, luteolin, *β*-sitosterol, and kaempferol were the main active ingredients of QGD acting on osteosarcoma, and major signaling pathways such as PI3K-Akt signaling pathway, MAPK signaling pathway, and IL-17 signaling pathway were observed. TP53, SRC, and ESR1 were identified as key proteins that docked well with their regulated compounds.

**Conclusion:**

QGD is effective against osteosarcoma through multicomponent, multitarget, and multipathway. This study was helpful for finding effective targets and compounds for osteosarcoma treatment.

## 1. Introduction

Osteosarcoma is the most common primary bone solid malignant tumor. Local pain, followed by localized swelling and limitation of joint movement, is the typical sign and symptom of osteosarcoma [1]. Adolescence is the highest incidence rate of osteosarcoma. The majority of cases occur in children and adolescents aged 10 to 30, with 10% occurring in those over the age of 60 [2]. Distant metastasis can be detected in approximately 15% to 20% of patients at the initial diagnosis, with the lung being the most common metastatic site, accounting for about 85% of metastatic diseases [3]. The 5-year survival rate for localized osteosarcoma is 67%, compared with only 20% in metastatic patients, which is the leading cause of death in patients with osteosarcoma [4]. Although new targeted drugs and immune drugs have been widely used in clinics, the survival rate of osteosarcoma has not been significantly improved. Therefore, it is of great value to better understand the metastasis mechanism of osteosarcoma and find appropriate targets and drugs to prolong the survival time of osteosarcoma.

Numerous studies have shown that traditional Chinese medicine (TCM) can not only alleviate the symptoms of tumor patients, such as fatigue, chronic pain, and cachexia, but also improve their quality of life and reduce the adverse reactions and complications caused by chemotherapy, radiotherapy, and targeted therapy [5]. In China, TCM treatment runs through the treatment of tumors.

In TCM, osteosarcoma is classified as “osteoma,” “osteomyelitis,” and “indurated knee mass.” It is believed that osteosarcoma is frequently caused by insufficient endowment, a lack of genuine qi. Pathogenic qi enters the body, traveling with meridian qi and blood to the bone marrow, causing qi and blood stagnation, meridian obstruction, tendon erosion, and bone formation. On this basis, we established the “pulmonary deficiency phlegm obstruction syndrome” osteosarcoma pulmonary metastasis rat model and found that Qingfei Gujin Decoction (QGD, composed of Astragalus membranaceus, Fritillaria thunbergii, Platycodon grandiflorum, Hedyotis diffusa, and Coicis semen) has a good antiosteosarcoma pulmonary metastasis effect, and its mechanism may be related to the downregulation of IL-10, TGF-*β*1, and CXCR4 expressions. [6]

**Figure 1:**
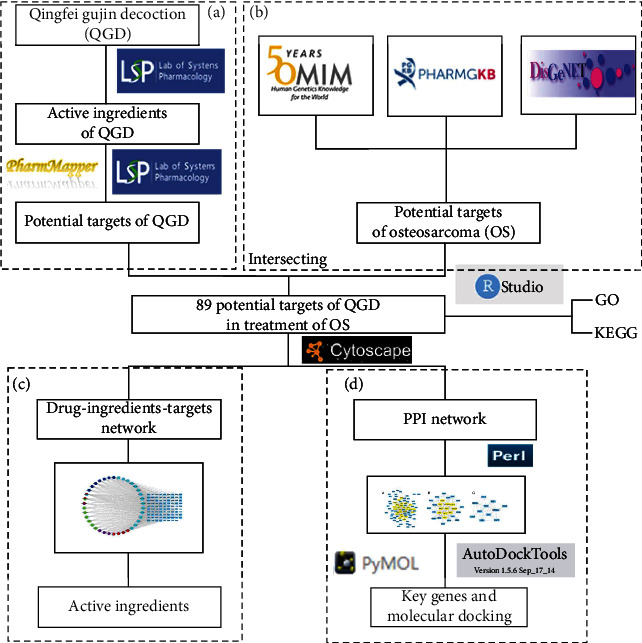
The flowchart of network pharmacology and molecular docking. (a) The screening of active ingredients of QGD. (b) Target collection for QGD. (c) Drug-ingredient-target network construction and active ingredients. (d) PPI network and key gene molecular docking.

Network pharmacology, a new concept in TCM research, has been widely used to study complex network relationships between TCM and diseases. To better understand the potential mechanism of QGD in the treatment of osteosarcoma, we used network pharmacology and molecular docking technology to screen the active components of QGD and predict its possible targets and pathways in osteosarcoma, with the goal of providing a theoretical foundation for the treatment of osteosarcoma with TCM (Figure 1).

## 2. Materials and Methods

### 2.1. The Screening of Active Ingredients of QGD

Traditional Chinese Medicine Systems Pharmacology Database and Analysis Platform (https://old.tcmsp-e.com/tcmsp.php) is a one-of-a-kind pharmacological platform of Chinese herbal medicines that captures the relationships between drugs, targets, and diseases [7]. We collected the potential active ingredients using the following criteria: oral bioavailability (OB) ≥ 30%, drug like (DL) ≥ 0.18, and half-life (HL) ≥ 4H.

### 2.2. Target Collection for QGD

TCMSP was used to collect targets that interact with active ingredients. Simultaneously, the predicted targets of QGD were obtained using PharmMapper (http://www.lilab-ecust.cn/pharmmapper/), an online tool for identifying potential target candidates based on molecular structure [8]. All the molecular structures (mol2 format) of active ingredients were achieved from TCMSP.

### 2.3. Target Collection for Osteosarcoma

Online Mendelian Inheritance in Man (OMIM) (https://omim.org/) is a comprehensive, authoritative compendium of human genes. PharmGKB (https://www.pharmgkb.org) is created by the National Institutes of Health (NIH), which provides information about how human genetic variation affects response to medications [9]. DisGeNET (https://www.disgenet.org) is a versatile platform that can be used for different research purposes including the investigation of the molecular underpinnings of human diseases and their comorbidities, the analysis of the properties of disease genes, the generation of hypothesis on drug therapeutic action and drug adverse effects, the validation of computationally predicted disease genes, and the evaluation of text-mining method performance [10]. Candidate targets of osteosarcoma were gathered from OMIM, PharmGKB, and DisGeNET. We used the keywords “Osteosarcoma” and “Metastatic osteosarcoma” to search these databases. We chose genes with a score > 0.04 from the DisGeNET. To remove repeated genes and normalize the gene information, RStudio and the Practical Extraction and Report Language (Perl) were used.

### 2.4. Drug-Ingredient-Target Network Construction and PPI

“Venn” package was used to analyze the intersection targets between QGD component targets and disease targets, which were considered to be the potential targets of QGD in the treatment of osteosarcoma. The drug-ingredient-target network was constructed by Cytoscape software (version 3.2.1). In this network, the active ingredients were represented by circular nodes of various colors, while potential targets were represented by rectangular nodes.

STRING (https://cn.string-db.org/) is an online database for searching known protein interactions. The PPI network was constructed by importing the potential genes into the search tool to retrieve interacting genes, and the organism type was selected as Homo sapiens (humans). The minimum required interaction score was set with a medium confidence = 0.9, and all other parameters were left at their default values. The “string interactions. tsv” file was downloaded in order to visualize the network and determine the intersection of the PPI network and core genes.

The obtained “string interactions. tsv” file was imported into Cytoscape software, and any duplicated edges were removed. Betweenness centrality (BC), closeness centrality (CC), degree centrality (DC), eigenvector centrality (EC), and local average connectivity-based method (LAC) were calculated using the “CytoNCA.” Firstly, the top 50% candidate genes of all values are to be subnetworks. Secondly, the candidate genes with the top 50% of values in subnetworks are selected as critical genes and established core networks.

### 2.5. Enrichment Analysis

R packages including “colorspace,” “stringi,” and “ggplot2” were installed in RStudio, and a Bioconductor package that includes “DOSE,” “clusterProfiler,” and “enrichplot” was used for GO and Kyoto Encyclopedia of Genes and Genomes (KEGG) enrichment analyses.

### 2.6. Molecular Docking

AutoDockTools 1.5.6 was used to dock the key targets and their conditioning ingredients. The mol2 chemical structure of ingredients was downloaded from the TCMSP database, and all compounds were saved as ligand parameter files in pdbqt format. The Research Collaboratory for Structural Bioinformatics Protein Data Bank (RCSB PDB, http://rcsb.org), the US data center for the global PDB archive, makes PDB data freely available to all users in support of a “Structural View of Biology” [11]. In this docking process, the 3D structure of key targets was retrieved from RCSB PDB, and solvents and water molecules were removed from target protein receptor molecules using PyMOL software. Binding energy was used as a docking score to evaluate the protein-ligand binding potential of molecular docking. Results with value ≤ −5 were selected and considered to have moderate binding potential and tight combination.

## 3. Results

### 3.1. The Main Active Ingredients and Potential Targets of QGD

38 active ingredients were selected from TCMSP database. Mol ID, molecule names, OB, DL, and HL are displayed in Table 1. After summarizing the active component targets of TCMPS and the predicted targets of PharmMapper, 526 potential targets were obtained by running Perl, eliminating duplicate values, and converting symbols.

### 3.2. The Common Potential Targets of QGD and Osteosarcoma

**Figure 2:**
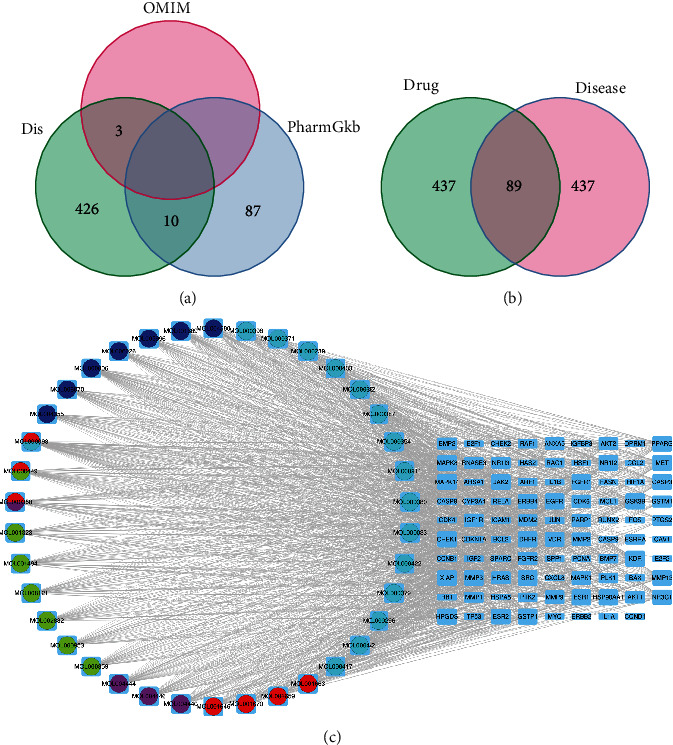
Potential targets and drug-ingredient-target network. (a) All the potential targets of OS. (b) Intersecting genes between drug and disease. (c) Drug-ingredient-target network of QGD acting on OS.

A total of 526 potential targets of osteosarcoma were collected, including 3 from OMIM, 97 from PharmGKB, and 439 from DisGeNET (Figure 2(a)). Finally, a total of 89 intersecting genes were screened as candidate targets to further research (Figure 2(b)).

### 3.3. Drug-Ingredient-Target Network and PPI

The drug-ingredient-target network was visualized using Cytoscape software. Figure 1(c) shows Hedyotis diffusa in red, Fritillaria thunbergii in rose red, Coicis semen in green, Astragalus membranaceus in light blue, and Platycodon grandiflorum in dark blue. According to supplement table 1, quercetin was associated with 77 potential targets, luteolin with 56 potential targets, and *β*-sitosterol and kaempferol with 51 potential targets, which may be the main active ingredient of QGD for the treatment of osteosarcoma. In Figure 1(c), the components were represented by the circle, and the candidate targets were represented by the square. The greater the size of the shape, the more the components associated with it. KDR, SRC, MAPK14, HPGDS, GSK3B, MET, MMP3, HSP90AA1, GSTP1, FGFR1, ESR1, EGFR, DHFR, CHEK1, and CASP3 are found to be the most frequently associated with active ingredients.

**Figure 3:**
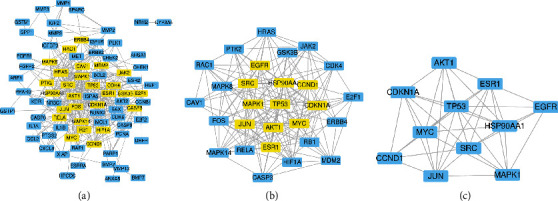
The screening of core network. (a) The original PPI network. (b) Subnetwork. (c) The core network.

89 candidate genes were introduced into STRING. According to the screening conditions, the “string interactions. tsv” was imported into Cytoscape to establish the PPI network. The network was consisted of 82 nodes and 400 edges. The genes with BC, CC, EC, DC, and LAC greater than the median were screened to construct the subnetwork, which included 28 nodes and 175 edges. According to the final values of BC, CC, EC, DC, and LAC (Table 2), 11 targets were obtained to become the core network, and TP53, SRC, and ESR1 were considered as the key genes (Figure 3).

### 3.4. Go Enrichment Analysis

**Figure 4:**
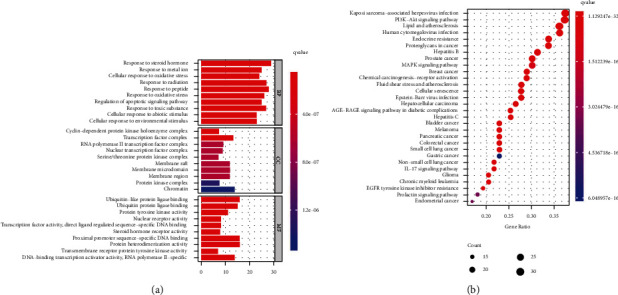
Enrichment analysis on 89 potential targets. (a) The barplot of GO enrichment. (b) The KEGG bubble.

GO enrichment includes biological process (BP), cellular component (CC), and molecular function (MF). 89 potential targets of QGD in the treatment of osteosarcoma were analyzed by R package. As shown in Figure 4(a), BP mainly included response to steroid hormones (go: 0048545), response to metal ions (go: 0010038), cell response to oxidative stress (go: 0034599), response to radiation (go: 0009314), response to peptides (go: 1901652), response to oxidative stress (go: 0006979), regulation of apoptosis signal pathway (go: 2001233), response to toxic substances (go: 0009636), cell response to abiotic stimuli (go: 0071214), and cell response to environmental stimuli (go: 0104004). In terms of CC, it mainly included chromatin (go: 0000785), transcription factor complex (go: 0005667), membrane raft (go: 0045121), membrane microregion (go: 0098857), membrane region (go: 0098589), RNA polymerase II transcription factor complex (go: 0090575), nuclear transcription factor complex (go: 0044798), cyclin-dependent protein kinase holoenzyme complex (go: 0000307), serine/threonine protein kinase complex (go: 1902554), and protein kinase complex (go: 1902911); MF mainly included ubiquitin-like protein ligase binding (go: 0044389), proximal promoter sequence-specific DNA binding (go: 0000987), protein heterodimerization activity (go: 0046982), ubiquitin protein ligase binding (go: 0031625), DNA binding transcription activator activity, RNA polymerase II specificity (go: 0001228), protein tyrosine kinase activity (go: 0004713), nuclear receptor activity (go: 0004879) transcription factor activity, direct ligand-regulated sequence-specific DNA binding (go: 0098531), steroid hormone receptor activity (go: 0003707), and transmembrane receptor protein tyrosine kinase activity (go: 0004714). Based on this, QGD in the treatment of osteosarcoma might be the result of multiple mechanisms.

### 3.5. KEGG Pathway Enrichment Analysis

In order to further explore the possible mechanism of QGD in the treatment of osteosarcoma, we performed KEGG pathway enrichment analysis on 89 target targets. As shown in Figure 4(b), the main related pathways included PI3K Akt signal pathway, proteoglycan in cancer, MAPK signal pathway, chemical carcinogenesis receptor activation pathway, cell aging, IL-17 signal pathway, and EGFR tyrosine kinase inhibitor resistance.

### 3.6. Molecular Docking Results

**Figure 5:**
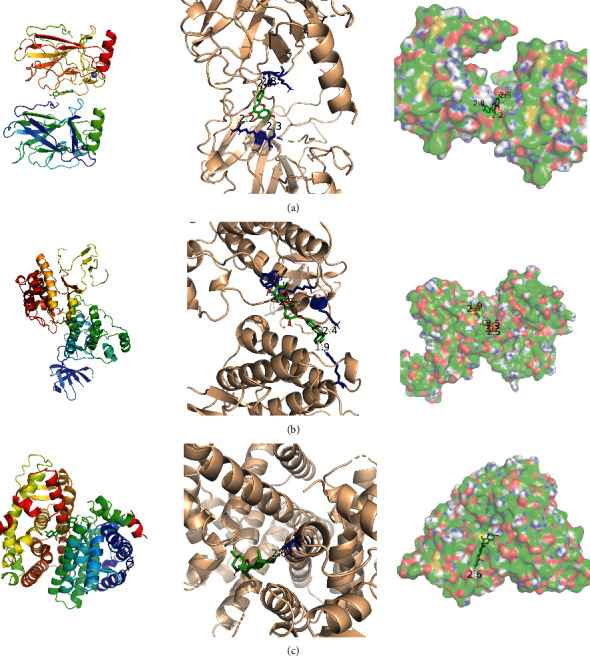
Molecular docking results. (a) Structure with an initial ligand of luteolin (MOL000006) with TP53 (PDB ID: 5o1h). (b) Structure with an initial ligand of robinin (MOL006070) with SRC (PDB ID: 1yoj). (c) Structure with an initial ligand of peimisine (MOL004440) with ESR1 (PDB ID: 4tuz).

Essential genes were selected for molecular docking with compounds that might regulate these targets (Supplement Table 2). The results showed that the docking binding energy of the key targets and the active ingredients was basically lower than -5 kcal/mol. Taking the lowest binding energy for example, the results can be seen in Table 3 and the structural diagrams are shown in Figure 5.

## 4. Discussion

It is a global problem to prevent postoperative recurrence and distant metastasis of osteosarcoma. Neoadjuvant chemotherapy, surgery, and postoperative adjuvant chemotherapy are the accepted standard modalities for the treatment of localized osteosarcoma. High-dose methotrexate (HD-MTX), cisplatin (DDP), adriamycin (ADM), ifosfamide (IFO), epirubicin (EPI), and etoposide (VP-16) are commonly used for osteosarcoma chemotherapy [12]. The MAP regimen composed of HD-MTX, DDP, and ADM is the standard regimen of most treatment centers in Europe and America. For high-grade osteosarcoma patients without metastasis, studies have shown that neoadjuvant chemotherapy combined with limb salvage surgery improved postoperative limb function and long-term quality of life [13]. The effectiveness of chemotherapy is evaluated by means of histological analysis of tumor necrosis, known as “the Huvos score” [14]. Studies have shown that 5-year disease-free survival (DFS) and overall survival (OS) are associated with the rate of histological necrosis of chemotherapy [15, 16]. Pathological fractures account for about 17% of bone tumors in children [17]. Meta-analysis confirmed that pathological fractures of osteosarcoma were associated with poor OS and event-free survival (EFS), but not with local recurrence [18]. On the contrary, Salunke et al. believed that pathological fracture was a negative prognostic indicator of osteosarcoma and might be associated with a lower 5-year EFS rate and a higher local recurrence rate [19]. The third generation of nitrogen-containing bisphosphonates, such as zoledronic acid, had been shown to reduce osteolysis caused by bone metastasis, but the role in inhibiting pulmonary metastasis remained controversial [20]. Lung metastasis occurs in 80-90% of patients after surgery. Chemotherapy and targeted drug therapy are first recommended for osteosarcoma patients with lung metastasis, then according to the treatment results to decide limb salvage or amputation surgery. Although clinical trials have been conducted with anti-PD 1 drug and anti-PD-L1 drug, no surprising results have been observed in osteosarcoma (Table 4). In addition, chemotherapy resistance is one of the reasons for osteosarcoma therapeutic failure. Several universal mechanisms of acquired resistance have been discovered, such as drug transport, drug metabolism, and epithelial-mesenchymal transition, which provide new methods for future treatment strategies to improve the prognosis of osteosarcoma [21]. Personalized medicine, including targeted therapies as well as immunotherapy, offers new possibilities to counteract resistance to conventional treatments for patients with cancer [22].

TCM has the benefits of safety and low toxicity, and it is increasingly being used for the prevention and treatment of osteosarcoma patients in China after surgery and chemotherapy. Network pharmacology has evolved into a sophisticated method for studying the practical components and complex mechanisms of TCM and compound prescriptions. In this study, the active ingredients of QGD were collected and network pharmacology was used to validate the effect of multitarget and multichannel treatment.

QGD, composed of Astragalus membranaceus, Fritillaria thunbergii, Platycodon grandiflorum, Hedyotis diffusa, and Coicis semen, has been shown to effectively prevent local recurrence and metastasis after osteosarcoma surgery. According to our study, the main active ingredients of QGD acting on osteosarcoma may be quercetin, luteolin, *β*-sitosterol, and kaempferol. Quercetin was a flavonol compound with a variety of biological activities which had been widely used to treat cardiovascular diseases, diabetes, tumors, and other diseases. Dana et al. hypothesized that quercetin inhibited osteosarcoma cell proliferation, migration, and invasion, inducing autophagy and apoptosis, and could play a separate or synergistic role in overcoming drug resistance in osteosarcoma cell lines [45]. Luteolin was a kind of natural flavonoid found in many plants and had anti-inflammatory, antiallergic, antiviral, and antitumor properties. In osteosarcoma, luteolin was involved in inhibiting tumor cell proliferation, inducing tumor cell apoptosis, affecting tumor cell cycle distribution, and inhibiting tumor angiogenesis and could be used as a tumor apoptosis sensitizer or antioxidant [46]. Meanwhile, it had been reported that luteolin could be acted as an enhancer to sensitize doxorubicin-mediated autophagy signaling in osteosarcoma cells [47]. *β*-Sitosterol was one of the most common phytosterols. A meta-analysis revealed that consuming high levels of dietary *β*-sitosterol might have a positive effect on antitumor mechanisms [48]. Although the mechanism of *β*-sitosterol in osteosarcoma was rarely reported, it has been shown to improve bone fragility and fracture occurrence [49]. Kaempferol had the effects of antioxidant, anti-inflammatory, proapoptotic, cardioprotective, and anticancer activities. In 2010, it was reported that kaempferol reduced the cell viabilities of osteosarcoma cells in a dose-dependent manner and induced apoptosis in human osteosarcoma cells via endoplasmic reticulum stress mitochondrial signaling pathways [50]. Chen et al. further confirmed that kaempferol attenuated the MAPK signaling pathways including ERK, JNK, and p38, resulting in decreased DNA binding ability of AP-1, and, hence, the downregulation of the expression and enzymatic activities of MMP-2, MMP-9, and Upa, contributing to the inhibition of metastasis of osteosarcoma cells [51].

P53 was encoded by the TP53 gene on human chromosome 17 and served as a tumor suppressor gene in the human body, protecting genomic integrity. Mutation in the TP53 gene was detectable in about 50% of patients with tumor, and more than 75% of TP53 gene mutations resulted in a loss of wild-type p53 activities, thereby promoting tumorigenesis, progression, and metastasis [52]. TP53 mutations were found in 47% to 90% of patients with osteosarcoma, and TP53 patients with mutations had poor overall survival rates [53]. Data suggested that TP53 mutations had a negative impact on 2-year overall survival [54]. Therefore, targeting TP53 may be an effective strategy in the treatment of osteosarcoma in the future. SRC family kinases were the most prominent family of nonreceptor tyrosine kinases. As the oldest oncogene, SRC was one of the best-studied targets for cancer therapy, which was closely related to regulating appreciation, angiogenesis, invasion metastasis, and bone metabolism [55]. Src could be activated by multiple signaling pathways to become phospho-Src (p-Src), and researchers had found that the expression of Src and p-Src in osteosarcoma was significantly higher, and the expression level was related to the clinical stage, tumor metastasis, and survival time of osteosarcoma, which could be used as an auxiliary index to judge the malignant phenotype of osteosarcoma and prompt the prognosis of osteosarcoma [56]. The Src pathway was associated with osteosarcoma metastasis. About 95% of samples examined express Src or had evidence of downstream activation of this pathway. As a potent selective SRC kinase inhibitor, the clinical trials had shown that saracatinib (AZD0530) was well tolerated but had no apparent impact on overall survival of osteosarcoma [57]. ESR1, a gene that encodes estrogen receptor *α* (ER), had been widely studied in breast cancer. Although there were fewer studies on ESR1 and osteosarcoma, ESR1-mediated estrogen effects associated with bone mineralization have been reported [58]. One of the mechanisms associated with the poor prognosis of osteosarcoma was abnormal ESR1 methylation [59]. Our study found that the core genes of QGD in the treatment of osteosarcoma were TP53, SRC, and ESR1, and molecular docking revealed that the binding capacity of the core gene to its regulated compounds was lower than -5 kcal/mol, suggesting that QGD could be multitargeted for osteosarcoma.

PI3K/AKT was one of the most important carcinogenic pathways in human cancer, and it was frequently overactivated in osteosarcoma. It was involved in tumor occurrence and progression, including proliferation, invasion, cell cycle progression, angiogenesis, and chemical resistance. Therefore, targeting the PI3K/AKT signaling pathway was proposed as a potential treatment for osteosarcoma [60]. The MAPK signaling pathway was involved in a variety of cellular processes, and its downstream pathways were JNK, P38, and ERK. In osteosarcoma, Xue et al. found that iron chelators could activate the ROS-related MAPK signaling pathway, promoting apoptosis and reducing malignant proliferation [61]. According to Zhang et al., the Chinese herb cardamomin might inhibit the proliferation, migration, and invasion of osteosarcoma by activating the P38 and MAPK signaling pathways [62]. Interleukin 17 was a CD4+ T cell-derived cytokine that stimulated some tumor cells to secrete angiogenesis factor, and the IL-17 receptor might represent a marker for the osteosarcoma metastasis [63]. By enriching potential targets through KEGG, we found that PI3K/AKT, MAPK, and IL-17 signaling pathways were the main pathways of QGD in the treatment of osteosarcoma, indicating that QGD could inhibit the invasion and metastasis of osteosarcoma through multiple pathways.

In summary, QGD had a variety of components and played an antiosteosarcoma role via multiple targets and pathways. Unfortunately, the efficacy of QGD in the treatment of osteosarcoma had not been validated at the molecular level, and further research was required to supplement the conclusion. At the same time, through network pharmacology and molecular docking technology, the role of Qingfei Gujin Decoction in different stages of osteosarcoma cannot be clearly defined. Its dose, intervention time, and exact curative effect need to be further studied in the future.

## 5. Conclusion

A total of 38 active ingredients were extracted from the QGD. 526 targets and 526 disease targets were collected, as well as 89 common targets were generated. Quercetin, luteolin, *β*-sitosterol, and kaempferol were the main active ingredients. We discovered that the main pathways of QGD against osteosarcoma were the PI3K-Akt signaling pathway, proteoglycans in cancer, MAPK signaling pathway, chemical carcinogenesis receptor activation pathway, cell aging, IL-17 signaling pathway, and EGFR tyrosine kinase inhibitor resistance. In addition, TP53, SRC, and ESR1 were presumed to be key proteins because of their good docking with the regulated compounds. These findings may aid in the identification of effective targets and compounds for osteosarcoma treatment, as well as provide a particular reference for osteosarcoma treatment.

## Figures and Tables

**Table 1:** The information of 38 active ingredients.

Mol ID	Molecule name	OB%	DL	HL
MOL001663	(4aS,6aR,6aS,6bR,8aR,10R,12aR,14bS)-10-hydroxy-2,2,6a,6b,9,9,12a-heptamethyl-1,3,4,5,6,6a,7,8,8a,10,11,12,13,14b-tetradecahydropicene-4a-carboxylic acid	32.0280133	0.75713	4.337611
MOL001659	Poriferasterol	43.8298516	0.75596	5.341727
MOL001670	2-Methoxy-3-methyl-9,10-anthraquinone	37.8277056	0.20517	28.820662
MOL001646	2,3-Dimethoxy-6-methyl anthraquinone	34.8586047	0.26255	28.975305
MOL000211	Mairin	55.3770734	0.7761	8.873708
MOL000239	Jaranol	50.8288168	0.29148	15.50148
MOL000296	Hederagenin	36.9139058	0.75072	5.347511
MOL000033	(3S,8S,9S,10R,13R,14S,17R)-10,13-dimethyl-17-[(2R,5S)-5-propan-2-yloctan-2-yl]-2,3,4,7,8,9,11,12,14,15,16,17-dodecahydro-1H-cyclopenta[a]6henanthrene-3-ol	36.2284706	0.78288	5.217923
MOL000354	Isorhamnetin	49.6043771	0.306	14.339263
MOL000371	3,9-Di-O-methylnissolin	53.7415267	0.47573	8.996019
MOL000379	9,10-Dimethoxypterocarpan-3-O-*β*-D-glucoside	36.736688	0.9243	13.063156
MOL000380	(6aR,11aR)-9,10-dimethoxy-6a,11a-dihydro-6H-benzofurano[3,2-c]chromen-3-ol	64.2554545	0.42486	8.493699
MOL000387	Bifendate	31.0978239	0.66553	17.961941
MOL000392	Formononetin	69.6738806	0.21202	17.036852
MOL000398	Isoflavanone	109.986656	0.29572	15.507494
MOL000417	Calycosin	47.7518278	0.24278	17.096724
MOL000422	Kaempferol	41.8822495	0.24066	14.743371
MOL000433	FA	68.9604362	0.7057	24.811237
MOL000442	1,7-Dihydroxy-3,9-dimethoxy pterocarpene	39.0454111	0.47943	7.946297
MOL000098	Quercetin	46.4333481	0.27525	14.400548
MOL004355	Spinasterol	42.9793655	0.75534	5.321195
MOL001689	Acacetin	34.9735727	0.24082	17.248472
MOL000006	Luteolin	36.1626293	0.24552	15.944492
MOL004580	Cis-dihydroquercetin	66.4369979	0.27344	14.513484
MOL005996	2-O-methyl-3-O-*β*-D-glucopyranosyl platycogenate A	45.1502334	0.25226	6.025842
MOL006026	Dimethyl 2-O-methyl-3-O-a-D-glucopyranosyl platycogenate A	39.2075766	0.25368	5.037639
MOL006070	Robinin	39.8437311	0.70731	16.672864
MOL001323	Sitosterol alpha1	43.2812704	0.78354	5.640765
MOL001494	Mandenol	41.9962005	0.19321	5.385969
MOL002882	[(2R)-2,3-dihydroxypropyl] (Z)-octadec-9-enoate	34.1310776	0.29824	5.189662
MOL000359	Sitosterol	36.9139058	0.7512	5.371091
MOL000449	Stigmasterol	43.8298516	0.75665	5.574595
MOL008121	2-Monoolein	34.2349738	0.29162	4.41187
MOL000953	CLR	37.8738975	0.67677	4.518834
MOL000358	Beta-sitosterol	36.9139058	0.75123	5.355491
MOL004440	Peimisine	57.4023933	0.8055	14.39177
MOL004444	Ziebeimine	64.2465779	0.70486	7.809565
MOL004446	6-Methoxyl-2-acetyl-3-methyl-1,4-naphthoquinone-8-O-beta-D-glucopyranoside	33.3073438	0.57257	31.005736

**Table 2:** The final values of BC, CC, EC, DC, and LAC in core network.

Name	Betweenness	Closeness	Degree	Eigenvector	LAC
TP53	5.521429	1	10	0.371665	6.4
SRC	4.771429	0.909091	9	0.336607	5.555556
ESR1	4.169048	0.909091	9	0.340247	5.777778
HSP90AA1	2.935714	0.833333	8	0.308101	5.25
MYC	2.438095	0.833333	8	0.309834	5.5
JUN	2.002381	0.833333	8	0.313054	5.25
MAPK1	1.535714	0.769231	7	0.279498	5.142857
CDKN1A	1.269048	0.769231	7	0.277749	4.857143
AKT1	0.821429	0.769231	7	0.288141	4.857143
CCND1	0.535714	0.714286	6	0.248775	4.333333
EGFR	0	0.666667	5	0.20902	4

**Table 3:** The lowest binding energy of key targets.

Target and PDB ID	Compound	Compound 2D structure	Grid box size	Affinity (kcal/mol)
TP53 (5o1h)	MOL000006	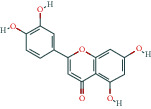	40^∗^40^∗^40	-8.1
SRC (1yoj)	MOL006070	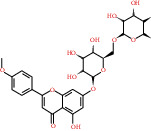	60^∗^60^∗^60	-10.4
ESR1 (4tuz)	MOL004440	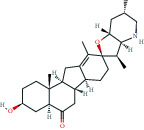	50^∗^60^∗^50	-10.4

**Table 4:** Common treatment of osteosarcoma.

Low-grade osteosarcoma	Surgical therapy		

High-grade osteosarcoma	Localized disease	Neoadjuvant chemotherapy/chemotherapy	AP (DDP-ADM) [23]
			MAP (HD-MTX, DDP, ADM) [24]
			IAP (IFO, DDP, ADM) [25]
			M-EI (MTX-etoposide-IFO) [26]
		Recurrent/refractory/metastatic	Pirarubicin-DDP [27]
			Paclitaxel-doxorubicin [28]
			Cyclophosphamide-VP-16 [29]
			Gemcitabine-docetaxel [30]
			Pemetrexed [31]
	Lung metastasis	Removable metastases	Neoadjuvant chemotherapy-extensive resection-excision of metastases
		Unresectable metastases	Chemotherapy and (or) radiotherapy
		Immunotherapy	IL-2 [32]
			Chimeric antigen receptor T cells (CAR-T) [33]
			Pembrolizumab [34]
			Camrelizumab [35]
			Durvalumab-tremelimumab [36]
		Molecular targeted therapy	Sorafenib [37]
			Sorafenib-everolimus [38]
			Regorafenib [39]
			Cabozantinib [40]
			Anlotinib [41]
			Apatinib [42]
		Antiangiogenic drugs	Endostar [43]
			Bevacizumab [44]

## Data Availability

The raw data supporting the conclusions of this manuscript will be made available by the authors, without undue reservation, to any qualified researcher.

## Supplementary Materials

Supplementary 1 Supplement table 1: active ingredients and corresponding targets.

Supplementary 2 Supplement table 2: the molecular docking results between the key targets and the active ingredients.
