# Supplementary material for: Research of the Active Components and Potential Mechanisms of Qingfei Gujin Decoction in the Treatment of Osteosarcoma Based on Network Pharmacology and Molecular Docking Technology
Source: Comput Math Methods Med. 2022 Nov 23;2022:7994425. doi: 10.1155/2022/7994425 (PMC9713469; doi:10.1155/2022/7994425)
Supplement: Supplementary 1 — Supplement table 1: active ingredients and corresponding targets. [file 7994425.f1.docx]

Supplement table 1 Active ingredients and corresponding targets

| MOL ID | Target | MOL ID | Target | MOL ID | Target | MOL ID | Target | MOL ID | Target | MOL ID | Target |
| --- | --- | --- | --- | --- | --- | --- | --- | --- | --- | --- | --- |
| MOL000006 | PTGS2 | MOL000098 | CCND1 | MOL000211 | MMP3 | MOL000354 | PARP1 | MOL000380 | PTGS2 | MOL001659 | MAPK14 |
| MOL000006 | HSP90AA1 | MOL000098 | BCL2 | MOL000211 | MMP9 | MOL000354 | MMP1 | MOL000380 | HSP90AA1 | MOL001659 | XIAP |
| MOL000006 | RELA | MOL000098 | FOS | MOL000211 | NR1I2 | MOL000354 | AKT1 | MOL000380 | ESR2 | MOL001659 | PLK1 |
| MOL000006 | EGFR | MOL000098 | CDKN1A | MOL000211 | NR1I3 | MOL000354 | AKT2 | MOL000380 | MAPK14 | MOL001659 | GSK3B |
| MOL000006 | AKT1 | MOL000098 | BAX | MOL000211 | PPARG | MOL000354 | BMP7 | MOL000380 | GSK3B | MOL001659 | KDR |
| MOL000006 | CCND1 | MOL000098 | CASP9 | MOL000211 | HPGDS | MOL000354 | CASP3 | MOL000380 | KDR | MOL001659 | FGFR2 |
| MOL000006 | CDKN1A | MOL000098 | MMP2 | MOL000211 | SRC | MOL000354 | CDK6 | MOL000380 | FGFR2 | MOL001659 | ESRRA |
| MOL000006 | CASP9 | MOL000098 | MMP9 | MOL000211 | VDR | MOL000354 | DHFR | MOL000380 | MET | MOL001659 | PARP1 |
| MOL000006 | MMP2 | MOL000098 | MAPK1 | MOL000211 | XIAP | MOL000354 | EGFR | MOL000380 | MMP1 | MOL001659 | IGF1R |
| MOL000006 | MMP9 | MOL000098 | RB1 | MOL000239 | PTGS2 | MOL000354 | ERBB4 | MOL000380 | AKT1 | MOL001659 | VDR |
| MOL000006 | MAPK1 | MOL000098 | JUN | MOL000239 | ESR2 | MOL000354 | PTK2 | MOL000380 | AKT2 | MOL001659 | SRC |
| MOL000006 | RB1 | MOL000098 | AHSA1 | MOL000239 | HSP90AA1 | MOL000354 | FGFR1 | MOL000380 | ANXA5 | MOL001659 | HPGDS |
| MOL000006 | CDK4 | MOL000098 | CASP3 | MOL000239 | CHEK1 | MOL000354 | NR3C1 | MOL000380 | BMP2 | MOL001659 | PPARG |
| MOL000006 | JUN | MOL000098 | TP53 | MOL000239 | MAPK14 | MOL000354 | GSTP1 | MOL000380 | CASP3 | MOL001659 | NR1I3 |
| MOL000006 | CASP3 | MOL000098 | CASP8 | MOL000239 | ARF1 | MOL000354 | IGF1R | MOL000380 | CDK6 | MOL001659 | NR1I2 |
| MOL000006 | TP53 | MOL000098 | RAF1 | MOL000239 | PLK1 | MOL000354 | JAK2 | MOL000380 | CHEK1 | MOL001659 | MMP3 |
| MOL000006 | MDM2 | MOL000098 | MMP1 | MOL000239 | GSK3B | MOL000354 | MET | MOL000380 | DHFR | MOL001659 | MMP2 |
| MOL000006 | MMP1 | MOL000098 | HIF1A | MOL000239 | FGFR2 | MOL000354 | MAPK8 | MOL000380 | EGFR | MOL001659 | MMP13 |
| MOL000006 | PCNA | MOL000098 | HSPA5 | MOL000239 | RNASE3 | MOL000354 | MMP13 | MOL000380 | ERBB4 | MOL001659 | MAPK8 |
| MOL000006 | ERBB2 | MOL000098 | ERBB2 | MOL000239 | PARP1 | MOL000354 | MMP3 | MOL000380 | FGFR1 | MOL001659 | MAPK1 |
| MOL000006 | PPARG | MOL000098 | CYP3A4 | MOL000239 | MMP1 | MOL000354 | MMP9 | MOL000380 | NR3C1 | MOL001659 | MET |
| MOL000006 | ICAM1 | MOL000098 | CAV1 | MOL000239 | AKT1 | MOL000354 | NR1I2 | MOL000380 | GSTM1 | MOL001659 | MDM2 |
| MOL000006 | MCL1 | MOL000098 | MYC | MOL000239 | ANXA5 | MOL000354 | HPGDS | MOL000380 | GSTP1 | MOL001659 | JAK2 |
| MOL000006 | CCNB1 | MOL000098 | ICAM1 | MOL000239 | CASP3 | MOL000354 | HRAS | MOL000380 | JAK2 | MOL001659 | HSP90AA1 |
| MOL000006 | XIAP | MOL000098 | IL1B | MOL000239 | CDK6 | MOL000354 | SRC | MOL000380 | MAPK1 | MOL001659 | GSTP1 |
| MOL000006 | MET | MOL000098 | CCL2 | MOL000239 | DHFR | MOL000354 | VDR | MOL000380 | MAPK8 | MOL001659 | NR3C1 |
| MOL000006 | ESR2 | MOL000098 | CXCL8 | MOL000239 | EGFR | MOL000354 | KDR | MOL000380 | MMP13 | MOL001659 | FGFR1 |
| MOL000006 | MAPK14 | MOL000098 | NR1I2 | MOL000239 | ERBB4 | MOL000354 | XIAP | MOL000380 | MMP2 | MOL001659 | ESR1 |
| MOL000006 | PLK1 | MOL000098 | CCNB1 | MOL000239 | ESR1 | MOL000358 | PTGS2 | MOL000380 | MMP3 | MOL001659 | ERBB4 |
| MOL000006 | GSK3B | MOL000098 | IL1A | MOL000239 | FGFR1 | MOL000358 | HSP90AA1 | MOL000380 | MMP9 | MOL001659 | EGFR |
| MOL000006 | RNASE3 | MOL000098 | HAS2 | MOL000239 | NR3C1 | MOL000358 | OPRM1 | MOL000380 | NR1I2 | MOL001659 | DHFR |
| MOL000006 | PARP1 | MOL000098 | PARP1 | MOL000239 | GSTM1 | MOL000358 | BCL2 | MOL000380 | PPARG | MOL001659 | CHEK1 |
| MOL000006 | KDR | MOL000098 | NR1I3 | MOL000239 | GSTP1 | MOL000358 | BAX | MOL000380 | HPGDS | MOL001659 | CASP3 |
| MOL000006 | VDR | MOL000098 | CHEK2 | MOL000239 | IGF1R | MOL000358 | CASP9 | MOL000380 | RAC1 | MOL001659 | BMP2 |
| MOL000006 | SRC | MOL000098 | HSF1 | MOL000239 | JAK2 | MOL000358 | JUN | MOL000380 | RAF1 | MOL001659 | ANXA5 |
| MOL000006 | HRAS | MOL000098 | SPP1 | MOL000239 | MET | MOL000358 | CASP3 | MOL000380 | HRAS | MOL001659 | AKT2 |
| MOL000006 | RAF1 | MOL000098 | RUNX2 | MOL000239 | MAPK8 | MOL000358 | CASP8 | MOL000380 | SRC | MOL001663 | ESR2 |
| MOL000006 | HPGDS | MOL000098 | E2F1 | MOL000239 | MMP13 | MOL000358 | AKT1 | MOL000380 | VDR | MOL001663 | MAPK14 |
| MOL000006 | NR1I2 | MOL000098 | E2F2 | MOL000239 | MMP3 | MOL000358 | ANXA5 | MOL000380 | XIAP | MOL001663 | GSK3B |
| MOL000006 | MMP3 | MOL000098 | IGFBP3 | MOL000239 | MMP9 | MOL000358 | BMP2 | MOL000387 | PTGS2 | MOL001663 | KDR |
| MOL000006 | MMP13 | MOL000098 | IGF2 | MOL000239 | NR1I2 | MOL000358 | CDK6 | MOL000387 | KDR | MOL001663 | FGFR2 |
| MOL000006 | MAPK8 | MOL000098 | GSTM1 | MOL000239 | PPARG | MOL000358 | CHEK1 | MOL000387 | MET | MOL001663 | PARP1 |
| MOL000006 | JAK2 | MOL000098 | ESR2 | MOL000239 | HPGDS | MOL000358 | DHFR | MOL000387 | HSP90AA1 | MOL001663 | MET |
| MOL000006 | IGF1R | MOL000098 | MAPK14 | MOL000239 | RAC1 | MOL000358 | EGFR | MOL000387 | MAPK14 | MOL001663 | IGF1R |
| MOL000006 | GSTP1 | MOL000098 | XIAP | MOL000239 | RAF1 | MOL000358 | ERBB4 | MOL000387 | RNASE3 | MOL001663 | HSP90AA1 |
| MOL000006 | GSTM1 | MOL000098 | ARF1 | MOL000239 | HRAS | MOL000358 | ESR1 | MOL000387 | MMP1 | MOL001663 | MMP1 |
| MOL000006 | NR3C1 | MOL000098 | PLK1 | MOL000239 | SRC | MOL000358 | FGFR1 | MOL000387 | AKT1 | MOL001663 | BMP2 |
| MOL000006 | FGFR1 | MOL000098 | GSK3B | MOL000239 | VDR | MOL000358 | NR3C1 | MOL000387 | CASP3 | MOL001663 | PPARG |
| MOL000006 | PTK2 | MOL000098 | RNASE3 | MOL000239 | KDR | MOL000358 | GSTM1 | MOL000387 | CDK6 | MOL001663 | MAPK8 |
| MOL000006 | ESR1 | MOL000098 | KDR | MOL000239 | XIAP | MOL000358 | GSTP1 | MOL000387 | CHEK1 | MOL001663 | MAPK1 |
| MOL000006 | ERBB4 | MOL000098 | VDR | MOL000296 | PTGS2 | MOL000358 | IGF1R | MOL000387 | DHFR | MOL001663 | MMP13 |
| MOL000006 | DHFR | MOL000098 | SRC | MOL000296 | ESR2 | MOL000358 | JAK2 | MOL000387 | EGFR | MOL001663 | GSTP1 |
| MOL000006 | CHEK1 | MOL000098 | HRAS | MOL000296 | MAPK14 | MOL000358 | MAPK1 | MOL000387 | ESR1 | MOL001663 | NR1I3 |
| MOL000006 | CDK6 | MOL000098 | HPGDS | MOL000296 | PLK1 | MOL000358 | MAPK8 | MOL000387 | FGFR1 | MOL001663 | EGFR |
| MOL000006 | BMP7 | MOL000098 | MMP13 | MOL000296 | GSK3B | MOL000358 | MMP13 | MOL000387 | NR3C1 | MOL001663 | SRC |
| MOL000006 | AKT2 | MOL000098 | MAPK8 | MOL000296 | KDR | MOL000358 | MMP2 | MOL000387 | GSK3B | MOL001663 | ANXA5 |
| MOL000033 | ESR2 | MOL000098 | MET | MOL000296 | FGFR2 | MOL000358 | MMP3 | MOL000387 | GSTM1 | MOL001663 | ESR1 |
| MOL000033 | MAPK14 | MOL000098 | JAK2 | MOL000296 | ESRRA | MOL000358 | MMP9 | MOL000387 | GSTP1 | MOL001663 | CHEK1 |
| MOL000033 | PLK1 | MOL000098 | IGF1R | MOL000296 | PARP1 | MOL000358 | NR1I2 | MOL000387 | JAK2 | MOL001663 | MDM2 |
| MOL000033 | GSK3B | MOL000098 | GSTP1 | MOL000296 | MET | MOL000358 | MMP1 | MOL000387 | MMP3 | MOL001663 | DHFR |
| MOL000033 | KDR | MOL000098 | NR3C1 | MOL000296 | IGF1R | MOL000358 | MET | MOL000387 | MMP9 | MOL001663 | MMP3 |
| MOL000033 | FGFR2 | MOL000098 | FGFR1 | MOL000296 | AKT2 | MOL000358 | RNASE3 | MOL000387 | NR1I2 | MOL001663 | ERBB4 |
| MOL000033 | ESRRA | MOL000098 | PTK2 | MOL000296 | ANXA5 | MOL000358 | FGFR2 | MOL000387 | PPARG | MOL001663 | HPGDS |
| MOL000033 | PARP1 | MOL000098 | ESR1 | MOL000296 | BMP2 | MOL000358 | KDR | MOL000387 | HPGDS | MOL001663 | NR3C1 |
| MOL000033 | IGF1R | MOL000098 | ERBB4 | MOL000296 | CASP3 | MOL000358 | GSK3B | MOL000387 | RAC1 | MOL001663 | NR1I2 |
| MOL000033 | AKT2 | MOL000098 | DHFR | MOL000296 | CHEK1 | MOL000358 | PPARG | MOL000387 | RAF1 | MOL001663 | MMP2 |
| MOL000033 | ANXA5 | MOL000098 | CHEK1 | MOL000296 | DHFR | MOL000358 | HPGDS | MOL000387 | HRAS | MOL001663 | CASP3 |
| MOL000033 | BMP2 | MOL000098 | CDK6 | MOL000296 | EGFR | MOL000358 | MAPK14 | MOL000387 | SRC | MOL001663 | VDR |
| MOL000033 | CASP3 | MOL000098 | BMP7 | MOL000296 | ERBB4 | MOL000358 | ESR2 | MOL000387 | VDR | MOL001663 | FGFR1 |
| MOL000033 | CHEK1 | MOL000098 | AKT2 | MOL000296 | ESR1 | MOL000358 | RAC1 | MOL000387 | XIAP | MOL001663 | XIAP |
| MOL000033 | DHFR | MOL000211 | ESR2 | MOL000296 | FGFR1 | MOL000358 | HRAS | MOL000392 | ESR1 | MOL001663 | JAK2 |
| MOL000033 | EGFR | MOL000211 | MAPK14 | MOL000296 | NR3C1 | MOL000358 | SRC | MOL000392 | PPARG | MOL001663 | CDK6 |
| MOL000033 | ERBB4 | MOL000211 | GSK3B | MOL000296 | GSTM1 | MOL000358 | VDR | MOL000392 | PTGS2 | MOL001663 | AKT1 |
| MOL000033 | ESR1 | MOL000211 | KDR | MOL000296 | GSTP1 | MOL000358 | XIAP | MOL000392 | ESR2 | MOL001663 | MMP9 |
| MOL000033 | FGFR1 | MOL000211 | FGFR2 | MOL000296 | HSP90AA1 | MOL000358 | ESRRA | MOL000392 | MAPK14 | MOL001663 | GSTM1 |
| MOL000033 | NR3C1 | MOL000211 | ESRRA | MOL000296 | JAK2 | MOL000358 | PARP1 | MOL000392 | GSK3B | MOL001663 | HRAS |
| MOL000033 | GSTP1 | MOL000211 | PARP1 | MOL000296 | MDM2 | MOL000358 | NR1I3 | MOL000392 | HSP90AA1 | MOL001670 | ESR1 |
| MOL000033 | HSP90AA1 | MOL000211 | IGF1R | MOL000296 | MAPK1 | MOL000358 | MDM2 | MOL000392 | CHEK1 | MOL001670 | PTGS2 |
| MOL000033 | JAK2 | MOL000211 | HSP90AA1 | MOL000296 | MAPK8 | MOL000358 | AKT2 | MOL000392 | JUN | MOL001670 | OPRM1 |
| MOL000033 | MDM2 | MOL000211 | MMP1 | MOL000296 | MMP13 | MOL000359 | ESR2 | MOL000392 | XIAP | MOL001670 | HSP90AA1 |
| MOL000033 | MET | MOL000211 | ANXA5 | MOL000296 | MMP2 | MOL000359 | MAPK14 | MOL000392 | KDR | MOL001670 | ESR2 |
| MOL000033 | MAPK1 | MOL000211 | BMP2 | MOL000296 | MMP3 | MOL000359 | GSK3B | MOL000392 | MET | MOL001670 | MAPK14 |
| MOL000033 | MAPK8 | MOL000211 | CASP3 | MOL000296 | NR1I2 | MOL000359 | KDR | MOL000392 | AKT1 | MOL001670 | GSK3B |
| MOL000033 | MMP13 | MOL000211 | CHEK1 | MOL000296 | NR1I3 | MOL000359 | FGFR2 | MOL000392 | CASP3 | MOL001670 | KDR |
| MOL000033 | MMP2 | MOL000211 | DHFR | MOL000296 | PPARG | MOL000359 | ESRRA | MOL000392 | DHFR | MOL001670 | FGFR2 |
| MOL000033 | MMP3 | MOL000211 | EGFR | MOL000296 | HPGDS | MOL000359 | PARP1 | MOL000392 | EGFR | MOL001670 | MET |
| MOL000033 | NR1I2 | MOL000211 | ERBB4 | MOL000296 | SRC | MOL000359 | MET | MOL000392 | ERBB4 | MOL001670 | MMP3 |
| MOL000033 | NR1I3 | MOL000211 | ESR1 | MOL000296 | VDR | MOL000359 | IGF1R | MOL000392 | FGFR1 | MOL001670 | BMP2 |
| MOL000033 | PPARG | MOL000211 | PTK2 | MOL000296 | XIAP | MOL000359 | VDR | MOL000392 | NR3C1 | MOL001670 | MAPK8 |
| MOL000033 | HPGDS | MOL000211 | FGFR1 | MOL000354 | ESR1 | MOL000359 | SRC | MOL000392 | GSTP1 | MOL001670 | ANXA5 |
| MOL000033 | SRC | MOL000211 | NR3C1 | MOL000354 | PPARG | MOL000359 | HPGDS | MOL000392 | IGF1R | MOL001670 | CASP3 |
| MOL000033 | VDR | MOL000211 | GSTM1 | MOL000354 | PTGS2 | MOL000359 | PPARG | MOL000392 | MDM2 | MOL001670 | CHEK1 |
| MOL000033 | XIAP | MOL000211 | GSTP1 | MOL000354 | ESR2 | MOL000359 | NR1I3 | MOL000392 | MAPK1 | MOL001670 | CDK6 |
| MOL000098 | PPARG | MOL000211 | JAK2 | MOL000354 | MAPK14 | MOL000359 | NR1I2 | MOL000392 | MAPK8 | MOL001670 | FGFR1 |
| MOL000098 | PTGS2 | MOL000211 | MDM2 | MOL000354 | GSK3B | MOL000359 | MMP3 | MOL000392 | MMP13 | MOL001670 | SRC |
| MOL000098 | HSP90AA1 | MOL000211 | MET | MOL000354 | HSP90AA1 | MOL000359 | MMP2 | MOL000392 | MMP3 | MOL001670 | EGFR |
| MOL000098 | MMP3 | MOL000211 | MAPK1 | MOL000354 | CHEK1 | MOL000359 | MMP13 | MOL000392 | MMP9 | MOL001670 | MMP13 |
| MOL000098 | RELA | MOL000211 | MAPK8 | MOL000354 | RELA | MOL000359 | MAPK8 | MOL000392 | NR1I2 | MOL001670 | NR1I3 |
| MOL000098 | EGFR | MOL000211 | MMP13 | MOL000354 | ARF1 | MOL000359 | MAPK1 | MOL000392 | HPGDS | MOL001670 | ERBB4 |
| MOL000098 | AKT1 | MOL000211 | MMP2 | MOL000354 | RNASE3 | MOL000392 | HRAS | MOL000392 | RAF1 | MOL001670 | GSTP1 |
| MOL000359 | MDM2 | MOL000392 | SRC | MOL000422 | ANXA5 | MOL000449 | PTGS2 | MOL001323 | MMP13 | MOL001670 | HPGDS |
| MOL000359 | JAK2 | MOL000392 | VDR | MOL000422 | BMP7 | MOL000449 | ESR2 | MOL001323 | MAPK8 | MOL001670 | DHFR |
| MOL000359 | HSP90AA1 | MOL000398 | MAPK14 | MOL000422 | CDK6 | MOL000449 | MAPK14 | MOL001323 | MAPK1 | MOL001670 | NR1I2 |
| MOL000359 | GSTP1 | MOL000398 | XIAP | MOL000422 | CHEK1 | MOL000449 | ARF1 | MOL001323 | MET | MOL001670 | XIAP |
| MOL000359 | NR3C1 | MOL000398 | GSK3B | MOL000422 | DHFR | MOL000449 | GSK3B | MOL001323 | MDM2 | MOL001670 | JAK2 |
| MOL000359 | FGFR1 | MOL000398 | KDR | MOL000422 | EGFR | MOL000449 | RNASE3 | MOL001323 | JAK2 | MOL001670 | MMP2 |
| MOL000359 | ESR1 | MOL000398 | RNASE3 | MOL000422 | ERBB4 | MOL000449 | PARP1 | MOL001323 | HSP90AA1 | MOL001670 | NR3C1 |
| MOL000359 | ERBB4 | MOL000398 | MMP1 | MOL000422 | ESR1 | MOL000449 | HSP90AA1 | MOL001323 | GSTP1 | MOL001670 | MAPK1 |
| MOL000359 | EGFR | MOL000398 | BMP7 | MOL000422 | ESR2 | MOL000449 | MMP1 | MOL001323 | GSTM1 | MOL001670 | VDR |
| MOL000359 | DHFR | MOL000398 | CASP3 | MOL000422 | PTK2 | MOL000449 | AKT1 | MOL001323 | NR3C1 | MOL001670 | MMP9 |
| MOL000359 | CHEK1 | MOL000398 | CDK6 | MOL000422 | FGFR1 | MOL000449 | AKT2 | MOL001323 | FGFR1 | MOL001670 | HRAS |
| MOL000359 | CDK6 | MOL000398 | CHEK1 | MOL000422 | NR3C1 | MOL000449 | BMP7 | MOL001323 | ESR1 | MOL001689 | PTGS2 |
| MOL000359 | CASP3 | MOL000398 | DHFR | MOL000422 | GSTP1 | MOL000449 | CASP3 | MOL001323 | ERBB4 | MOL001689 | HSP90AA1 |
| MOL000359 | BMP2 | MOL000398 | EGFR | MOL000422 | IGF1R | MOL000449 | CDK6 | MOL001323 | EGFR | MOL001689 | CHEK1 |
| MOL000359 | ANXA5 | MOL000398 | ESR1 | MOL000422 | JAK2 | MOL000449 | CHEK1 | MOL001323 | DHFR | MOL001689 | RELA |
| MOL000359 | AKT2 | MOL000398 | ESR2 | MOL000422 | MET | MOL000449 | DHFR | MOL001323 | CHEK1 | MOL001689 | BCL2 |
| MOL000371 | ESR1 | MOL000398 | PTK2 | MOL000422 | MMP13 | MOL000449 | EGFR | MOL001323 | CDK6 | MOL001689 | CDKN1A |
| MOL000371 | PTGS2 | MOL000398 | FGFR1 | MOL000422 | MMP3 | MOL000449 | ERBB4 | MOL001323 | CASP3 | MOL001689 | BAX |
| MOL000371 | OPRM1 | MOL000398 | NR3C1 | MOL000422 | MMP9 | MOL000449 | ESR1 | MOL001323 | BMP2 | MOL001689 | CASP3 |
| MOL000371 | AKT1 | MOL000398 | GSTM1 | MOL000422 | HPGDS | MOL000449 | PTK2 | MOL001323 | ANXA5 | MOL001689 | TP53 |
| MOL000371 | ANXA5 | MOL000398 | GSTP1 | MOL000422 | RAF1 | MOL000449 | FGFR1 | MOL001323 | AKT2 | MOL001689 | CASP8 |
| MOL000371 | BMP2 | MOL000398 | HSP90AA1 | MOL000422 | HRAS | MOL000449 | NR3C1 | MOL001323 | PTGS2 | MOL001689 | FASN |
| MOL000371 | CASP3 | MOL000398 | IGF1R | MOL000422 | SRC | MOL000449 | GSTP1 | MOL001494 | ESR2 | MOL001689 | MAPK14 |
| MOL000371 | CDK6 | MOL000398 | JAK2 | MOL000422 | VDR | MOL000449 | IGF1R | MOL001494 | MAPK14 | MOL001689 | XIAP |
| MOL000371 | CHEK1 | MOL000398 | MET | MOL000422 | KDR | MOL000449 | JAK2 | MOL001494 | XIAP | MOL001689 | ARF1 |
| MOL000371 | DHFR | MOL000398 | MAPK1 | MOL000422 | XIAP | MOL000449 | MET | MOL001494 | GSK3B | MOL001689 | KDR |
| MOL000371 | EGFR | MOL000398 | MAPK8 | MOL000433 | GSK3B | MOL000449 | MAPK8 | MOL001494 | KDR | MOL001689 | FGFR2 |
| MOL000371 | ERBB4 | MOL000398 | MMP13 | MOL000433 | ESR2 | MOL000449 | MMP13 | MOL001494 | FGFR2 | MOL001689 | RNASE3 |
| MOL000371 | FGFR1 | MOL000398 | MMP3 | MOL000433 | MAPK14 | MOL000449 | MMP3 | MOL001494 | ESRRA | MOL001689 | PARP1 |
| MOL000371 | NR3C1 | MOL000398 | MMP9 | MOL000433 | ARF1 | MOL000449 | MMP9 | MOL001494 | PARP1 | MOL001689 | MET |
| MOL000371 | GSTM1 | MOL000398 | NR1I2 | MOL000433 | PLK1 | MOL000449 | NR1I2 | MOL001494 | MET | MOL001689 | VDR |
| MOL000371 | GSTP1 | MOL000398 | PPARG | MOL000433 | MAPK8 | MOL000449 | HPGDS | MOL001494 | IGF1R | MOL001689 | SRC |
| MOL000371 | HSP90AA1 | MOL000398 | HPGDS | MOL000433 | RNASE3 | MOL000449 | HRAS | MOL001494 | VDR | MOL001689 | HRAS |
| MOL000371 | IGF1R | MOL000398 | RAC1 | MOL000433 | PARP1 | MOL000449 | SRC | MOL001494 | SRC | MOL001689 | RAF1 |
| MOL000371 | JAK2 | MOL000398 | RAF1 | MOL000433 | AKT1 | MOL000449 | VDR | MOL001494 | HPGDS | MOL001689 | RAC1 |
| MOL000371 | MAPK1 | MOL000398 | HRAS | MOL000433 | AKT2 | MOL000449 | KDR | MOL001494 | PPARG | MOL001689 | HPGDS |
| MOL000371 | MAPK8 | MOL000398 | SRC | MOL000433 | BMP7 | MOL000449 | XIAP | MOL001494 | NR1I3 | MOL001689 | PPARG |
| MOL000371 | MMP13 | MOL000398 | VDR | MOL000433 | CASP3 | MOL000449 | PLK1 | MOL001494 | NR1I2 | MOL001689 | NR1I2 |
| MOL000371 | MMP2 | MOL000417 | ESR1 | MOL000433 | CDK6 | MOL000449 | FGFR2 | MOL001494 | MMP9 | MOL001689 | MMP9 |
| MOL000371 | MMP3 | MOL000417 | PPARG | MOL000433 | CHEK1 | MOL000449 | ESRRA | MOL001494 | MMP3 | MOL001689 | MMP3 |
| MOL000371 | MMP9 | MOL000417 | PTGS2 | MOL000433 | DHFR | MOL000449 | PPARG | MOL001494 | MMP2 | MOL001689 | MMP2 |
| MOL000371 | NR1I2 | MOL000417 | ESR2 | MOL000433 | EGFR | MOL000449 | NR1I3 | MOL001494 | MMP13 | MOL001689 | MMP13 |
| MOL000371 | MMP1 | MOL000417 | MAPK14 | MOL000433 | ESR1 | MOL000449 | MMP2 | MOL001494 | MAPK8 | MOL001689 | MAPK8 |
| MOL000371 | MET | MOL000417 | GSK3B | MOL000433 | PTK2 | MOL000449 | MAPK1 | MOL001494 | MAPK1 | MOL001689 | JAK2 |
| MOL000371 | RNASE3 | MOL000417 | HSP90AA1 | MOL000433 | FGFR1 | MOL000449 | MDM2 | MOL001494 | MDM2 | MOL001689 | GSTP1 |
| MOL000371 | FGFR2 | MOL000417 | CHEK1 | MOL000433 | GSTM1 | MOL000449 | BMP2 | MOL001494 | JAK2 | MOL001689 | GSTM1 |
| MOL000371 | KDR | MOL000417 | XIAP | MOL000433 | GSTP1 | MOL000449 | ANXA5 | MOL001494 | HSP90AA1 | MOL001689 | GSK3B |
| MOL000371 | GSK3B | MOL000417 | MAPK8 | MOL000433 | HSP90AA1 | MOL000953 | ESR2 | MOL001494 | GSTP1 | MOL001689 | NR3C1 |
| MOL000371 | PPARG | MOL000417 | KDR | MOL000433 | IGF1R | MOL000953 | MAPK14 | MOL001494 | GSTM1 | MOL001689 | FGFR1 |
| MOL000371 | HPGDS | MOL000417 | RNASE3 | MOL000433 | JAK2 | MOL000953 | GSK3B | MOL001494 | NR3C1 | MOL001689 | ESR2 |
| MOL000371 | MAPK14 | MOL000417 | MMP1 | MOL000433 | MET | MOL000953 | KDR | MOL001494 | FGFR1 | MOL001689 | ESR1 |
| MOL000371 | ESR2 | MOL000417 | AKT1 | MOL000433 | MMP13 | MOL000953 | FGFR2 | MOL001494 | ESR1 | MOL001689 | ERBB4 |
| MOL000371 | RAC1 | MOL000417 | AKT2 | MOL000433 | MMP3 | MOL000953 | ESRRA | MOL001494 | ERBB4 | MOL001689 | EGFR |
| MOL000371 | HRAS | MOL000417 | CASP3 | MOL000433 | MMP9 | MOL000953 | PARP1 | MOL001494 | EGFR | MOL001689 | DHFR |
| MOL000371 | SRC | MOL000417 | CDK6 | MOL000433 | PPARG | MOL000953 | VDR | MOL001494 | DHFR | MOL001689 | CDK6 |
| MOL000371 | VDR | MOL000417 | DHFR | MOL000433 | HPGDS | MOL000953 | SRC | MOL001494 | CHEK1 | MOL001689 | AKT1 |
| MOL000371 | XIAP | MOL000417 | EGFR | MOL000433 | RAC1 | MOL000953 | HPGDS | MOL001494 | CASP3 | MOL002882 | ESR2 |
| MOL000379 | PTGS2 | MOL000417 | ERBB4 | MOL000433 | RAF1 | MOL000953 | PPARG | MOL001494 | BMP2 | MOL002882 | MAPK14 |
| MOL000379 | ESR2 | MOL000417 | FGFR1 | MOL000433 | HRAS | MOL000953 | NR1I3 | MOL001494 | ANXA5 | MOL002882 | KDR |
| MOL000379 | MAPK14 | MOL000417 | NR3C1 | MOL000433 | SPARC | MOL000953 | NR1I2 | MOL001494 | AKT2 | MOL002882 | FGFR2 |
| MOL000379 | ARF1 | MOL000417 | GSTM1 | MOL000433 | SRC | MOL000953 | MMP3 | MOL001494 | AKT1 | MOL002882 | RNASE3 |
| MOL000379 | PLK1 | MOL000417 | GSTP1 | MOL000433 | KDR | MOL000953 | MMP2 | MOL001494 | PTGS2 | MOL002882 | ESRRA |
| MOL000379 | KDR | MOL000417 | JAK2 | MOL000433 | XIAP | MOL000953 | MMP13 | MOL001646 | AKT1 | MOL002882 | PARP1 |
| MOL000379 | FGFR2 | MOL000417 | MET | MOL000442 | PTGS2 | MOL000953 | MAPK8 | MOL001646 | CASP3 | MOL002882 | IGF1R |
| MOL000379 | RNASE3 | MOL000417 | MMP13 | MOL000442 | HSP90AA1 | MOL000953 | MAPK1 | MOL001646 | CDK6 | MOL002882 | HSP90AA1 |
| MOL000379 | PARP1 | MOL000417 | MMP2 | MOL000442 | MAPK14 | MOL000953 | MET | MOL001646 | CHEK1 | MOL002882 | XIAP |
| MOL000379 | MET | MOL000417 | MMP3 | MOL000442 | PLK1 | MOL000953 | MDM2 | MOL001646 | DHFR | MOL002882 | VDR |
| MOL000379 | IGF1R | MOL000417 | MMP9 | MOL000442 | AKT1 | MOL000953 | JAK2 | MOL001646 | EGFR | MOL002882 | SRC |
| MOL000379 | HSP90AA1 | MOL000417 | NR1I2 | MOL000442 | AKT2 | MOL000953 | IGF1R | MOL001646 | ERBB4 | MOL002882 | HPGDS |
| MOL000379 | AKT1 | MOL000417 | HPGDS | MOL000442 | ANXA5 | MOL000953 | HSP90AA1 | MOL001646 | ESR1 | MOL002882 | PPARG |
| MOL000379 | AKT2 | MOL000417 | RAC1 | MOL000442 | BMP2 | MOL000953 | GSTP1 | MOL001646 | FGFR1 | MOL002882 | NR1I3 |
| MOL000379 | ANXA5 | MOL000417 | RAF1 | MOL000442 | CASP3 | MOL000953 | NR3C1 | MOL001646 | NR3C1 | MOL002882 | NR1I2 |
| MOL000379 | BMP2 | MOL000417 | HRAS | MOL000442 | CDK6 | MOL000953 | FGFR1 | MOL001646 | GSTM1 | MOL002882 | MMP9 |
| MOL000379 | BMP7 | MOL000417 | SRC | MOL000442 | CHEK1 | MOL000953 | ESR1 | MOL001646 | GSTP1 | MOL002882 | MMP3 |
| MOL000379 | CASP3 | MOL000417 | VDR | MOL000442 | DHFR | MOL000953 | ERBB4 | MOL001646 | HSP90AA1 | MOL002882 | MMP2 |
| MOL000379 | CDK6 | MOL000422 | PPARG | MOL000442 | EGFR | MOL000953 | EGFR | MOL001646 | IGF1R | MOL002882 | MMP13 |
| MOL000379 | CHEK1 | MOL000422 | PTGS2 | MOL000442 | ERBB4 | MOL000953 | DHFR | MOL001646 | JAK2 | MOL002882 | MAPK8 |
| MOL000379 | DHFR | MOL000422 | HSP90AA1 | MOL000442 | ESR1 | MOL000953 | CHEK1 | MOL001646 | MDM2 | MOL002882 | MAPK1 |
| MOL000379 | EGFR | MOL000422 | RELA | MOL000442 | ESR2 | MOL000953 | CDK6 | MOL001646 | MAPK1 | MOL002882 | MET |
| MOL000379 | ERBB4 | MOL000422 | AKT1 | MOL000442 | PTK2 | MOL000953 | CASP3 | MOL001646 | MAPK8 | MOL002882 | MDM2 |
| MOL000379 | ESR1 | MOL000422 | BCL2 | MOL000442 | FGFR1 | MOL000953 | BMP2 | MOL001646 | MMP13 | MOL002882 | JAK2 |
| MOL000379 | FGFR1 | MOL000422 | BAX | MOL000442 | NR3C1 | MOL000953 | ANXA5 | MOL001646 | MMP2 | MOL002882 | GSTP1 |
| MOL000379 | NR3C1 | MOL000422 | JUN | MOL000442 | GSK3B | MOL000953 | AKT2 | MOL001646 | MMP3 | MOL002882 | GSK3B |
| MOL000379 | GSK3B | MOL000422 | AHSA1 | MOL000442 | GSTM1 | MOL001323 | ESR2 | MOL001646 | MMP9 | MOL002882 | NR3C1 |
| MOL000379 | GSTP1 | MOL000422 | CASP3 | MOL000442 | GSTP1 | MOL001323 | MAPK14 | MOL001646 | NR1I2 | MOL002882 | FGFR1 |
| MOL000379 | JAK2 | MOL000422 | MAPK8 | MOL000442 | JAK2 | MOL001323 | GSK3B | MOL001646 | NR1I3 | MOL002882 | ESR1 |
| MOL000379 | MDM2 | MOL000422 | MMP1 | MOL000442 | MET | MOL001323 | KDR | MOL001646 | MET | MOL002882 | ERBB4 |
| MOL000379 | MAPK1 | MOL000422 | CYP3A4 | MOL000442 | MAPK8 | MOL001323 | FGFR2 | MOL001646 | RNASE3 | MOL002882 | EGFR |
| MOL000379 | MAPK8 | MOL000422 | ICAM1 | MOL000442 | MMP2 | MOL001323 | ESRRA | MOL001646 | GSK3B | MOL002882 | DHFR |
| MOL000379 | MMP13 | MOL000422 | NR1I2 | MOL000442 | MMP3 | MOL001323 | PARP1 | MOL001646 | PPARG | MOL002882 | CHEK1 |
| MOL000379 | MMP2 | MOL000422 | HAS2 | MOL000442 | MMP9 | MOL001323 | IGF1R | MOL001646 | HPGDS | MOL002882 | CDK6 |
| MOL000379 | MMP3 | MOL000422 | NR1I3 | MOL000442 | NR1I2 | MOL001323 | XIAP | MOL001646 | MAPK14 | MOL002882 | CASP3 |
| MOL000379 | NR1I2 | MOL000422 | GSTM1 | MOL000442 | PPARG | MOL001323 | VDR | MOL001646 | ESR2 | MOL002882 | BMP2 |
| MOL000379 | PPARG | MOL000422 | MAPK14 | MOL000442 | HPGDS | MOL001323 | SRC | MOL001646 | RAC1 | MOL002882 | ANXA5 |
| MOL000379 | HPGDS | MOL000422 | PLK1 | MOL000442 | RAC1 | MOL001323 | HPGDS | MOL001646 | HRAS | MOL002882 | AKT2 |
| MOL000379 | HRAS | MOL000422 | GSK3B | MOL000442 | HRAS | MOL001323 | PPARG | MOL001646 | SRC | MOL002882 | AKT1 |
| MOL000379 | SRC | MOL000422 | FGFR2 | MOL000442 | SRC | MOL001323 | NR1I3 | MOL001646 | VDR | MOL004355 | ESR2 |
| MOL000379 | VDR | MOL000422 | RNASE3 | MOL000442 | VDR | MOL001323 | NR1I2 | MOL001646 | KDR | MOL004355 | MAPK14 |
| MOL000379 | XIAP | MOL000422 | PARP1 | MOL000442 | KDR | MOL001323 | MMP3 | MOL001646 | XIAP | MOL004355 | PLK1 |
| MOL000380 | ESR1 | MOL000422 | AKT2 | MOL000442 | XIAP | MOL001323 | MMP2 | MOL001659 | ESR2 | MOL004355 | GSK3B |
| MOL004355 | PPARG | MOL004444 | ESRRA | MOL004580 | RNASE3 | MOL006026 | GSK3B | MOL008121 | ESR2 | MOL006070 | HRAS |
| MOL004355 | KDR | MOL004444 | PARP1 | MOL004580 | PARP1 | MOL006026 | KDR | MOL008121 | MAPK14 | MOL006070 | HPGDS |
| MOL004355 | FGFR2 | MOL004444 | MET | MOL004580 | MMP1 | MOL006026 | FGFR2 | MOL008121 | PLK1 | MOL006070 | PPARG |
| MOL004355 | ESRRA | MOL004444 | IGF1R | MOL004580 | XIAP | MOL006026 | RNASE3 | MOL008121 | KDR | MOL006070 | MMP9 |
| MOL004355 | PARP1 | MOL004444 | HSP90AA1 | MOL004580 | KDR | MOL006026 | ESRRA | MOL008121 | FGFR2 | MOL006070 | MMP3 |
| MOL004355 | IGF1R | MOL004444 | XIAP | MOL004580 | VDR | MOL006026 | PARP1 | MOL008121 | RNASE3 | MOL006070 | MMP2 |
| MOL004355 | XIAP | MOL004444 | VDR | MOL004580 | SRC | MOL006026 | XIAP | MOL008121 | ESRRA | MOL006070 | MMP13 |
| MOL004355 | VDR | MOL004444 | SRC | MOL004580 | HRAS | MOL006026 | VDR | MOL008121 | PARP1 | MOL006070 | MAPK8 |
| MOL004355 | SRC | MOL004444 | HPGDS | MOL004580 | RAF1 | MOL006026 | SRC | MOL008121 | IGF1R | MOL006070 | MAPK1 |
| MOL004355 | HPGDS | MOL004444 | PPARG | MOL004580 | HPGDS | MOL006026 | HRAS | MOL008121 | XIAP | MOL006070 | MET |
| MOL004355 | NR1I3 | MOL004444 | NR1I3 | MOL004580 | PPARG | MOL006026 | HPGDS | MOL008121 | VDR | MOL006070 | MDM2 |
| MOL004355 | NR1I2 | MOL004444 | NR1I2 | MOL004580 | NR1I2 | MOL006026 | PPARG | MOL008121 | SRC | MOL006070 | IGF1R |
| MOL004355 | MMP3 | MOL004444 | MMP3 | MOL004580 | MMP9 | MOL006026 | NR1I3 | MOL008121 | HPGDS | MOL006070 | GSTP1 |
| MOL004355 | MMP2 | MOL004444 | MMP2 | MOL004580 | MMP3 | MOL006026 | NR1I2 | MOL008121 | PPARG | MOL006070 | GSK3B |
| MOL004355 | MMP13 | MOL004444 | MMP13 | MOL004580 | MMP13 | MOL006026 | MMP9 | MOL008121 | NR1I3 | MOL006070 | FGFR1 |
| MOL004355 | MAPK8 | MOL004444 | MAPK8 | MOL004580 | MAPK8 | MOL006026 | MMP3 | MOL008121 | NR1I2 | MOL006070 | PTK2 |
| MOL004355 | MAPK1 | MOL004444 | MAPK1 | MOL004580 | MAPK1 | MOL006026 | MMP2 | MOL008121 | MMP9 | MOL006070 | ESR2 |
| MOL004355 | MET | MOL004444 | MDM2 | MOL004580 | MET | MOL006026 | MMP13 | MOL008121 | MMP3 | MOL006070 | ESR1 |
| MOL004355 | MDM2 | MOL004444 | JAK2 | MOL004580 | JAK2 | MOL006026 | MAPK8 | MOL008121 | MMP2 | MOL006070 | ERBB4 |
| MOL004355 | JAK2 | MOL004444 | GSTP1 | MOL004580 | IGF1R | MOL006026 | MAPK1 | MOL008121 | MMP13 | MOL006070 | EGFR |
| MOL004355 | HSP90AA1 | MOL004444 | GSTM1 | MOL004580 | GSTP1 | MOL006026 | MET | MOL008121 | MAPK8 | MOL006070 | DHFR |
| MOL004355 | GSTP1 | MOL004444 | NR3C1 | MOL004580 | GSTM1 | MOL006026 | MDM2 | MOL008121 | MAPK1 | MOL006070 | CHEK1 |
| MOL004355 | GSTM1 | MOL004444 | FGFR1 | MOL004580 | GSK3B | MOL006026 | JAK2 | MOL008121 | MET | MOL006070 | CDK6 |
| MOL004355 | NR3C1 | MOL004444 | ESR2 | MOL004580 | NR3C1 | MOL006026 | IGF1R | MOL008121 | MDM2 | MOL006070 | CASP3 |
| MOL004355 | FGFR1 | MOL004444 | ESR1 | MOL004580 | FGFR1 | MOL006026 | HSP90AA1 | MOL008121 | JAK2 | MOL006070 | BMP7 |
| MOL004355 | ESR1 | MOL004444 | ERBB4 | MOL004580 | PTK2 | MOL006026 | GSTP1 | MOL008121 | HSP90AA1 | MOL006070 | ANXA5 |
| MOL004355 | ERBB4 | MOL004444 | EGFR | MOL004580 | ESR1 | MOL006026 | NR3C1 | MOL008121 | GSTP1 | MOL006070 | AKT2 |
| MOL004355 | EGFR | MOL004444 | DHFR | MOL004580 | ERBB4 | MOL006026 | FGFR1 | MOL008121 | GSK3B | MOL006070 | AKT1 |
| MOL004355 | DHFR | MOL004444 | CHEK1 | MOL004580 | EGFR | MOL006026 | ESR1 | MOL008121 | NR3C1 | MOL005996 | ESR1 |
| MOL004355 | CHEK1 | MOL004444 | CDK6 | MOL004580 | DHFR | MOL006026 | ERBB4 | MOL008121 | FGFR1 | MOL005996 | ERBB4 |
| MOL004355 | CDK6 | MOL004444 | CASP3 | MOL004580 | CHEK1 | MOL006026 | EGFR | MOL008121 | ESR1 | MOL005996 | EGFR |
| MOL004355 | CASP3 | MOL004444 | BMP2 | MOL004580 | CDK6 | MOL006026 | DHFR | MOL008121 | ERBB4 | MOL005996 | DHFR |
| MOL004355 | BMP2 | MOL004444 | ANXA5 | MOL004580 | CASP3 | MOL006026 | CHEK1 | MOL008121 | EGFR | MOL005996 | CHEK1 |
| MOL004355 | ANXA5 | MOL004444 | AKT2 | MOL004580 | BMP7 | MOL006026 | CASP3 | MOL008121 | DHFR | MOL005996 | CASP3 |
| MOL004355 | AKT2 | MOL004446 | ESR1 | MOL004580 | AKT2 | MOL006026 | BMP7 | MOL008121 | CHEK1 | MOL005996 | BMP7 |
| MOL004440 | NR3C1 | MOL004446 | PTGS2 | MOL004580 | AKT1 | MOL006026 | BMP2 | MOL008121 | CDK6 | MOL005996 | BMP2 |
| MOL004440 | MAPK14 | MOL004446 | HSP90AA1 | MOL005996 | MAPK14 | MOL006026 | ANXA5 | MOL008121 | CASP3 | MOL005996 | ANXA5 |
| MOL004440 | XIAP | MOL004446 | MAPK14 | MOL005996 | GSK3B | MOL006026 | AKT1 | MOL008121 | BMP2 | MOL006026 | MAPK14 |
| MOL004440 | GSK3B | MOL004446 | FGFR2 | MOL005996 | KDR | MOL006070 | MAPK14 | MOL008121 | ANXA5 | MOL006026 | PLK1 |
| MOL004440 | KDR | MOL004446 | RNASE3 | MOL005996 | FGFR2 | MOL006070 | ARF1 | MOL008121 | AKT2 | MOL004446 | CHEK1 |
| MOL004440 | FGFR2 | MOL004446 | IGF1R | MOL005996 | RNASE3 | MOL006070 | PLK1 | MOL008121 | AKT1 | MOL004446 | CDK6 |
| MOL004440 | ESRRA | MOL004446 | MMP1 | MOL005996 | ESRRA | MOL006070 | FGFR2 | MOL004440 | DHFR | MOL004446 | CASP3 |
| MOL004440 | PARP1 | MOL004446 | XIAP | MOL005996 | PARP1 | MOL006070 | RNASE3 | MOL004440 | CHEK1 | MOL004446 | BMP7 |
| MOL004440 | HSP90AA1 | MOL004446 | KDR | MOL005996 | IGF1R | MOL006070 | PARP1 | MOL004440 | CASP3 | MOL004446 | AKT2 |
| MOL004440 | VDR | MOL004446 | SRC | MOL005996 | XIAP | MOL006070 | HSP90AA1 | MOL004440 | BMP2 | MOL004446 | AKT1 |
| MOL004440 | SRC | MOL004446 | HRAS | MOL005996 | SRC | MOL006070 | MMP1 | MOL004440 | ANXA5 | MOL004580 | PTGS2 |
| MOL004440 | HRAS | MOL004446 | RAF1 | MOL005996 | HRAS | MOL006070 | XIAP | MOL004440 | AKT2 | MOL004580 | HSP90AA1 |
| MOL004440 | HPGDS | MOL004446 | HPGDS | MOL005996 | HPGDS | MOL006070 | KDR | MOL004444 | MAPK14 | MOL004580 | ESR2 |
| MOL004440 | PPARG | MOL004446 | PPARG | MOL005996 | PPARG | MOL006070 | SRC | MOL004444 | PLK1 | MOL004580 | MAPK14 |
| MOL004440 | NR1I3 | MOL004446 | NR1I2 | MOL005996 | NR1I3 | MOL005996 | FGFR1 | MOL004444 | GSK3B | MOL004580 | PLK1 |
| MOL004440 | NR1I2 | MOL004446 | MMP9 | MOL005996 | NR1I2 | MOL005996 | PTK2 | MOL004444 | KDR | MOL004446 | EGFR |
| MOL004440 | MMP9 | MOL004446 | MMP3 | MOL005996 | MMP9 | MOL005996 | ESR2 | MOL004444 | FGFR2 | MOL004446 | DHFR |
| MOL004440 | MMP3 | MOL004446 | MMP2 | MOL005996 | MMP3 | MOL004440 | ERBB4 | MOL005996 | JAK2 | MOL004446 | GSTP1 |
| MOL004440 | MMP2 | MOL004446 | MMP13 | MOL005996 | MMP2 | MOL004440 | EGFR | MOL005996 | HSP90AA1 | MOL004446 | GSTM1 |
| MOL004440 | MMP13 | MOL004446 | MAPK8 | MOL005996 | MMP13 | MOL004440 | GSTP1 | MOL005996 | GSTP1 | MOL004446 | GSK3B |
| MOL004440 | MAPK8 | MOL004446 | MAPK1 | MOL005996 | MAPK8 | MOL004440 | FGFR1 | MOL005996 | NR3C1 | MOL004446 | FGFR1 |
| MOL004440 | MAPK1 | MOL004446 | MET | MOL005996 | MAPK1 | MOL004440 | ESR2 | MOL005996 | MDM2 | MOL004446 | ESR2 |
| MOL004440 | MET | MOL004446 | MDM2 | MOL005996 | MET | MOL004440 | ESR1 | MOL004440 | JAK2 | MOL004446 | JAK2 |
| MOL004440 | MDM2 |  |  |  |  |  |  |  |  |  |  |
