# Supplementary material for: Research of the Active Components and Potential Mechanisms of Qingfei Gujin Decoction in the Treatment of Osteosarcoma Based on Network Pharmacology and Molecular Docking Technology
Source: Comput Math Methods Med. 2022 Nov 23;2022:7994425. doi: 10.1155/2022/7994425 (PMC9713469; doi:10.1155/2022/7994425)
Supplement: Supplementary 2 — Supplement table 2: the molecular docking results between the key targets and the active ingredients. [file 7994425.f2.docx]

Supplement table 2 The molecular docking results between the key targets and the active ingredients

| Target | PDB ID | Ingredients | Affinity (kcal/mol) |
| --- | --- | --- | --- |
| TP53 | 5o1h | MOL000098 | -7.8 |
|  |  | MOL001689 | -7.6 |
|  |  | MOL000006 | -8.1 |
| SRC | 1yoj | MOL001646 | -7.7 |
|  |  | MOL001659 | -8.8 |
|  |  | MOL001663 | -8.5 |
|  |  | MOL001670 | -8.4 |
|  |  | MOL000449 | -9.1 |
|  |  | MOL000358 | -8.3 |
|  |  | MOL000098 | -7.9 |
|  |  | MOL000211 | -8.1 |
|  |  | MOL000239 | -7.9 |
|  |  | MOL000296 | -8.6 |
|  |  | MOL000033 | -9.0 |
|  |  | MOL000354 | -8.2 |
|  |  | MOL000371 | -7.7 |
|  |  | MOL000379 | -8.9 |
|  |  | MOL000380 | -7.1 |
|  |  | MOL000387 | -7.3 |
|  |  | MOL000392 | -7.7 |
|  |  | MOL000398 | -7.7 |
|  |  | MOL000417 | -7.9 |
|  |  | MOL000422 | -8.2 |
|  |  | MOL000433 | -8.2 |
|  |  | MOL000442 | -8.2 |
|  |  | MOL001689 | -8.2 |
|  |  | MOL004355 | -9.6 |
|  |  | MOL004580 | -7.7 |
|  |  | MOL005996 | -7.6 |
|  |  | MOL000006 | -8.2 |
|  |  | MOL006026 | -7.0 |
|  |  | MOL006070 | -10.4 |
|  |  | MOL001323 | -9.3 |
|  |  | MOL001494 | -4.7 |
|  |  | MOL002882 | -5.7 |
|  |  | MOL000359 | -9.2 |
|  |  | MOL008121 | -5.6 |
|  |  | MOL000953 | -8.4 |
|  |  | MOL004440 | -9.0 |
|  |  | MOL004444 | -8.5 |
|  |  | MOL004446 | -7.4 |
| ESR1 | 4tuz | MOL001646 | -8.7 |
|  |  | MOL001659 | -8.2 |
|  |  | MOL001663 | -8.8 |
|  |  | MOL001670 | -8.7 |
|  |  | MOL000449 | -9.0 |
|  |  | MOL000358 | -8.5 |
|  |  | MOL000098 | -8.4 |
|  |  | MOL000211 | -8.8 |
|  |  | MOL000239 | -7.5 |
|  |  | MOL000296 | -8.3 |
|  |  | MOL000033 | -8.3 |
|  |  | MOL000354 | -8.3 |
|  |  | MOL000371 | -7.5 |
|  |  | MOL000379 | -8.9 |
|  |  | MOL000380 | -8.1 |
|  |  | MOL000387 | -6.8 |
|  |  | MOL000392 | -7.7 |
|  |  | MOL000398 | -7.8 |
|  |  | MOL000417 | -7.6 |
|  |  | MOL000422 | -8.1 |
|  |  | MOL000433 | -8.1 |
|  |  | MOL000442 | -8.3 |
|  |  | MOL001689 | -7.5 |
|  |  | MOL004355 | -9.0 |
|  |  | MOL004580 | -7.6 |
|  |  | MOL005996 | -7.7 |
|  |  | MOL000006 | -8.0 |
|  |  | MOL006026 | -7.8 |
|  |  | MOL006070 | -9.3 |
|  |  | MOL001323 | -7.6 |
|  |  | MOL001494 | -4.4 |
|  |  | MOL002882 | -4.9 |
|  |  | MOL000359 | -7.8 |
|  |  | MOL008121 | -4.9 |
|  |  | MOL000953 | -8.2 |
|  |  | MOL004440 | -10.4 |
|  |  | MOL004444 | -10.3 |
|  |  | MOL004446 | -7.2 |
